# Reducing TGF‐β1 cooperated with StemRegenin 1 promoted the expansion ex vivo of cord blood CD34^+^ cells by inhibiting AhR signalling

**DOI:** 10.1111/cpr.12999

**Published:** 2021-01-31

**Authors:** Xuejun Zhu, Qihao Sun, Wen‐song Tan, Haibo Cai

**Affiliations:** ^1^ State Key Laboratory of Bioreactor Engineering East China University of Science and Technology Shanghai China

## Abstract

**Objective:**

As an inhibitor of the AhR signalling pathway, StemRegenin 1 (SR1) not only promotes the expansion of CD34^+^ cells but also increases CD34^−^ cell numbers. These CD34^−^ cells influenced the ex vivo expansion of CD34^+^ cells. In this work, the effects of periodically removing CD34^−^ cells combined with SR1 addition on the ex vivo expansion and biological functions of HSCs were investigated.

**Materials and methods:**

CD34^−^ cells were removed periodically with SR1 addition to investigate cell subpopulations, cell expansion, biological functions, expanded cell division mode and supernatant TGF‐β1 contents.

**Results:**

After 10‐day culture, the expansion of CD34^+^ cells in the CD34^−^ cell removal plus SR1 group was significantly higher than that in the control group and the SR1 group. Moreover, periodically removing CD34^−^ cells with SR1 addition improved the biological function of expanded CD34^+^ cells and significantly increased the percentage of self‐renewal symmetric division of CD34^+^ cells. In addition, the concentration of total TGF‐β1 and activated TGF‐β1 in the supernatant was significantly lower than those in the control group and the SR1 group. RT‐qPCR results showed that the periodic removal of CD34^−^ cells with cooperation from SR1 further reduced the expression of AhR‐related genes.

**Conclusions:**

Periodic removal of CD34^−^ cells plus cooperation with SR1 improved the expansion of CD34^+^ cells, maintained better biological function of expanded CD34^+^ cells and reduced the TGF‐β1 contents by downregulating AhR signalling.

## Graphical abstract

SR1 increased self‐renewal symmetric division of cord blood CD34^+^ cells. Removal of CD34^−^ cells cooperated with SR1 increased ex vivo expansion of cord blood CD34^+^ cells. Removal of CD34^−^ cells cooperated with SR1 further downregulated AhR signalling

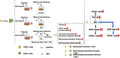

## 1 INTRODUCTION

With the ability of self‐renewal and differentiation, haematopoietic stem cells (HSCs) have great clinical value.^1^, ^2^, ^3^ Currently, ex vivo expansion of HSCs is the most effective way to solve the lack of HSCs. However, no breakthrough has been made in the ex vivo expansion of HSCs. The root cause for this is that the ex vivo regulation of the mode of HSC division is not sufficiently effective.

Self‐renewal symmetrical division, asymmetric division and differentiated symmetrical division are the three division modes of HSCs.^4^ During division, the cell fate determinant Numb is preferred to separate cells into differentiated daughter cells and is expressed at lower levels in stem cells,^5^ while mushisa‐2 (Msi2) is expressed at higher levels in stem cells and at lower levels in differentiated cells.^6^ In addition, the quantities of HSCs can be changed by regulating self‐renewal symmetrical division. Findings state that self‐renewal symmetrical division of HSCs is increased by attenuating Aryl hydrocarbon receptor (AhR) signalling in human HSCs in vivo.^7^ Therefore, regulation of AhR signalling may be beneficial to increase self‐renewal symmetrical division of HSCs.

AhR is an aromatic compound receptor and a family member of the basic helix‐loop‐helix‐period‐aryl hydrocarbon receptor nuclear translocator.^8^ Previous studies showed that activation of AhR accelerated the differentiation process of HSCs.^9^, ^10^, ^11^ Singh et al^12^ found that the exposure of the AhR activator 2,3,7,8‐tetrachlorodibenzo‐p‐dioxin (TCDD) exhibited diminished capacity to reconstitute and home to the marrow of irradiated recipients, and Laiosa et al^13^ reported that the long‐term self‐renewal ability of HSCs from TCDD‐exposed foetuses was decreased after initial reconstitution in mouse pregnancy. StemRegenin 1 (SR1) is an antagonist of AhR signalling and facilitates the expansion of HSCs.^14^, ^15^, ^16^ The expansion of hESC‐derived Lin^−^CD34^+^ haematopoietic progenitors was enhanced by SR1 in a concentration‐dependent manner.^14^ The number of CD34^+^, CD133^+^ and CD90^+^ haematopoietic stem and progenitor cells with SR1 was significantly increased in the culture of mouse peripheral blood CD34^+^ cells for 7 days.^15^ In summary, the inhibition of AhR signalling was beneficial to improve the expansion of HSCs.

CD34 is a key marker of HSCs. HSCs could lose the CD34 marker and differentiate into CD34^−^ cells during ex vivo expansion. These increased CD34^−^ cells influenced the expansion of CD34^+^ cells through secreting factors. Among these cytokines, transforming growth factor β1 (TGF‐β1) is a major cytokine that plays an inhibitory role in haematopoietic function, and the effect depends on the differentiation status of the cells.^17^, ^18^ High concentrations of TGF‐β1 strongly inhibited the expansion of HSPCs.^19^, ^20^, ^21^ The proliferation and differentiation of CD34^+^CD38^−^ cells and CD34^+^CD38^+^ cells were strongly suppressed with TGF‐β1 addition.^22^ Moreover, different mechanisms have been proposed to explain AhR/TGF‐β crosstalk in different cells.^23^, ^24^, ^25^, ^26^ Moyer et al^24^ demonstrated that TGF‐β1 was an endogenous activator of AHR in a dose‐responsive manner in H1 L7.5c3 cells. However, TGF‐β1 decreased the expression of AhR mRNA in human HepG2 cells^27^ and Staršíchová et al^25^ found that TGF‐β1 inhibited AhR‐mediated gene expression through inhibition of AhR expression and downregulation of nuclear AhR in the human nontumorigenic prostate epithelial cell line BPH‐1. The relationship between AhR signalling and TGF‐β in HSCs is not clear.

Therefore, to investigate the effects of periodically removing CD34^−^ cells with SR1 addition on the ex vivo expansion of CD34^+^ cells, enrichment of CD34^+^ cells was performed on day 4 and day 7 to reduce CD34^−^ cells and the TGF‐β1 content. The cell expansion, biological function of expanded CD34^+^ cells, division mode of CD34^+^ cells and concentration of TGF‐β1 in the supernatant were explored. It was found that removal of CD34^−^ cells plus addition of SR1 increased the expansion of CD34^+^ cells and maintained better physiological function of expanded CD34^+^ cells by inhibiting AhR signalling. These results are expected to provide support for optimizing the haematopoietic stem cell culture process ex vivo.

## 2 MATERIALS AND METHODS

### 2.1 Cell preparation

Cord blood CD34^+^ cells were obtained by density gradient centrifugation with Ficoll/Histopaque and enriched by a CD34‐positive immunomagnetic bead EasySep™ sorting system (Miltenyi Biotec). The purity of enriched CD34^+^ cells detected by flow cytometry (FACSCalibur, Becton Dickinson) reached >95%.

### 2.2 Cell culture

Fresh CD34+ cells (5.0 × 10^4^ cells/mL) were seeded in each well of 48‐well plates (Nunc) with 1 mL serum‐free medium (StemSpan^®^ ‐SFEM, Stemcell). The serum‐free medium was supplemented with 50 ng/mL SCF, 20 ng/mL TPO, 50 ng/mL FL and 20 ng/mL IL‐6 (PeproTech). Cells were cultured at 37°C in an incubator containing 5% CO_2_ and 21% O_2_. The serum‐free medium was refreshed by half volume on day 4 and day 7.

To determine the optimal SR1 addition strategy, 0.5 μmol/L SR1 was added, as shown in Table 1. Samples were taken, and the numbers of total cells were counted. The relative expansion fold ratio was calculated to evaluate the expansion of CD34^+^ cells.

$$\text{Fold}\;\text{expansion}\;\text{of}\;\text{total}\;\text{cells}=\frac{\text{the}\;\text{number}\;\text{of}\;\text{total}\;\text{cells}}{\text{the}\;\text{number}\;\text{on}\;\text{day}\;0}$$

$$\text{Relative}\;\text{expansion}\;\text{fold}\;\text{ratio}=\frac{\text{the}\;\text{expansion}\;\text{fold}\;\text{of}\;T}{\text{the}\;\text{expansion}\;\text{fold}\;\text{of}\;\text{control}}$$

**TABLE 1:** SR1 addition strategy

Group	Addition frequency of 0.5 μmol/L SR1
Control	Without SR1
T1	12 h^−1^
T2	24 h^−1^
T3	48 h^−1^
T4	96 h^−1^

To investigate the effects of removing CD34^−^ cells with SR1 addition on the expansion of CD34^+^ cells, experiments were performed as shown in Table 2.

**TABLE 2:** Experimental design

Group	Culture method
Control	Half medium was refreshed on day 4 and day 7
SR1	0.5 μmol/L SR1 was added at the frequency of 12 h^−1^; half medium was refreshed on day 4 and day 7
Removal of CD34^−^ cells + SR1	0.5 μmol/L SR1 was added at the frequency of 12 h^−1^, CD34^−^ cells were removed with Human Cord Blood Positive Selection Kit II (Stemcell), and isolated CD34^+^ cells were reseeded with fresh medium on day 4 and day 7

### 2.3 Cell subpopulation analysis by flow cytometry

A total of 1 × 10^6^ cells were stained with mouse anti‐human antibodies (BD) for 30 minutes at 4°C in the dark and analysed with flow cytometry (FACSCalibur, BD) to determine the cell subpopulation in culture. These antibodies included PE‐CD34, BV421‐CD34, FITC‐CD38, APC‐CD38, BV510‐CD45A, PerCP‐Cy5.5‐CD90 and PE‐CD49f. According to the proportions in total cells, the numbers of CD34^+^ cells and CD34^+^CD38^−^ cells were calculated, and the fold expansion was calculated using the numbers divided by the numbers on day 0.

### 2.4 Colony‐forming assay

Expanded CD34^+^ cells (500 cells/mL) were reseeded in each well of 48‐well plates with 300 μL semisolid IMDM medium containing 0.9% methylcellulose (Gibco), 50 ng/mL SCF, 20 ng/mL G‐CSF, 14 ng/mL GM‐CSF, 20 ng/mL IL‐3, 20 ng/mL IL‐6 and 2 U/mL EPO. Cells were cultured at 37°C in a humidified incubator with 5% CO_2_ for 10‐14 days. Groups of cloned cells containing more than 50 cells were scored as colony‐forming units (CFUs), and different CFU types were identified according to the morphology of clones. The frequency of each CFU type and total CFUs were scored as the number of colonies per 10^4^ seeded cells. Types of CFUs included granulocyte, erythrocyte, macrophage, megakaryocyte common precursors (CFU‐GEMM), granulocyte/macrophage common precursors (CFU‐GM) and burst‐forming units for pure erythroid precursors (BFU‐E).

### 2.5 Secondary expansion assay of expanded CD34^+^ cells

Expanded CD34^+^ cells were separated using a Mini MACS magnetic separation system at the end of culture and were reseeded a density of 5 × 10^4^ cells/mL in 48‐well plates with 500 μL serum‐free medium in each well. The serum‐free medium was supplemented with 50 ng/mL SCF, 20 ng/mL TPO, and 50 ng/mL Flt3‐L. CD34^+^ cells were cultured for 14 days at 37°C in a humidified incubator with 5% CO_2_. The secondary expansion ability of CD34^+^ cells was evaluated based on the expansion of total cells and proportion of CD34^+^ cells on day 14.

### 2.6 Supernatant human TGF‐β1 concentration detection by ELISA

By centrifugation and removal of particulates, cell culture supernatants were collected to detect the concentration of TGF‐β1 by enzyme‐linked immunosorbent assay (ELISA) (R&D systems^®^).

### 2.7 Detection of Numb distribution by immunofluorescence staining

A total of 1 × 10^6^ cells were exposed to medium containing 75 nmol/L nocodazole (Sigma) for 22 h and transferred to fresh medium. Then, 1 × 10^6^ cells/mL cells were settled on poly‐l‐lysine‐coated coverslips (BD Biosciences), fixed with 4% paraformaldehyde (USB Corporation), permeabilized with Triton, blocked with 20% BSA (Invitrogen) for 30 minutes and incubated with primary antibody overnight at 4°C and secondary antibody for 1 hour at room temperature. Finally, cells were stained with DAPI (Sigma) to detect DNA. The division mode was identified using ImageJ according to the fluorescence distribution. Nocodazole interfered with the polymerization of microtubules and caused cell cycle arrest in the G2/M phase. The primary antibodies were rabbit anti‐Numb and goat anti‐CD34 (Abcam). Secondary antibodies were donkey anti‐rabbit lgG H&L Alexa Fluor^®^ 647 and donkey anti‐goat lgG H&L Alexa Fluor^®^ 488 (Abcam).

### 2.8 Detection of *numb* and *musashi2* by qRT‐PCR

RNA was isolated using a RNeasy Mini kit (Qiagen). cDNA was obtained from equal amounts of RNA using Superscript II reverse transcriptase (Invitrogen). Quantitative real‐time PCR was performed using iQ SYBR Green Supermix (Bio‐Rad) on a CFX 96 C1000™ Thermal cycler (Bio‐Rad). Primer sequences are listed in Table 3.

**TABLE 3:** Primers for genes used in RT‐PCR

Gene	Primer (5′‐3′)
B2M‐F	CCATCCGACATTGAAGTTGA
B2M‐R	TGGAGCAACCTGCTCACATA
GAPDH‐F	CCCTTTTGTAGGAGGGACT
GAPDH‐R	AATGCTTGCTGCTGCCTA
Numb‐F	CCTCAGCAGACAGGCATACA
Numb‐R	GGTTGGTAGGGGAGGGATTA
Msi2‐F	TGACATCGGTGCTCACTTCT
Msi2‐R	GCTATCTGGTGAGGTCTGCC
CYP1B1‐F	TTGACTCTGGAGTGGGAGTG
CYP1B1‐R	TCGGTGAGTGGCGTCAATTC
HSP90‐F	GGGCAACACCTCTACAAGGA
HSP90‐R	CTTGGGTCTGGGTTTCCTC
CEBP‐F	GGTGGACAAGAACAGCAACGAGTA
CEBP‐R	GGCGGTCATTGTCACTGGTCAG
PU.1‐F	GGCAACCGCAAGAAGATGACCTA
PU.1‐R	CACTTCGCCGCTGAACTGGTAG
Hoxa 9‐F	CCTGACTGACTATGCTTGTGGTTCTC
Hoxa 9‐R	TTGTCTCCGCCGCTCTCATTCT
MEIS1‐F	CGGCATCTACTCGTTCAGGAGGAA
MEIS1‐R	CATCACCTTGCTCACTGCTGTTGT
RUNX1‐F	ACTGTGATGGCTGGCAATGATGAA
RUNX1‐R	GCTCTGTGGTAGGTGGCGACTT
HOXB4‐F	AGGTCTTGGAGCTGGAGAAGGAAT
HOXB4‐R	GGTGTTGGGCAACTTGTGGTCTT
Tie2‐F	CTATCCGTTACAAGGTTCAAGGCAAGA
Tie2‐R	GCTGATACTGAGTGATGGTGGCATT
HES1‐F	CTGGAGAGGCGGCTAAGGTGTT
HES1‐R	CTGGAAGGTGACACTGCGTTGG
GATA2‐F	TGCGTCTCCAGCCTCATCTTCC
GATA2‐R	TTCCATCTTCATGCTCTCCGTCAGT
Stat5a‐F	TCCTGAACAACTGCTGCGTGATG
Stat5a‐R	TGCTGCCAACACTGAACTGAGAC
BMI1‐F	CAAGTTCACAAGACCAGACCACTACT
BMI1‐R	CATTGGCAGCATCAGCAGAAGGA
TGFBR1‐F	AGTCCAGCACTCTTGAGAGG
TGFBR1‐R	AAGAACGTTCGTGGTTCCGT
SMAD4‐F	TGTGTGACCTTTGGGCAAGT
SMAD4‐R	CTGCAAGGTAGCCAAGGTCA
P57^KIP2^‐F	TGAGCCAAGTGAGTACAGCG
P57^KIP2^‐R	TTGGGCTCTAAACTGCGAGG

### 2.9 Statistical analysis

The results are presented as scatter with the mean or mean ± standard deviation of the mean. A *t* test was used for statistical analysis between the two groups, and ANOVA was used for statistical analysis between three or more groups. *P* < .05 was considered statistically significant.

## 3 RESULTS

### 3.1 Periodic removal of CD34^−^ cells in cooperation with SR1 enhanced the expansion of CD34^+^ cells

**FIGURE 1:**
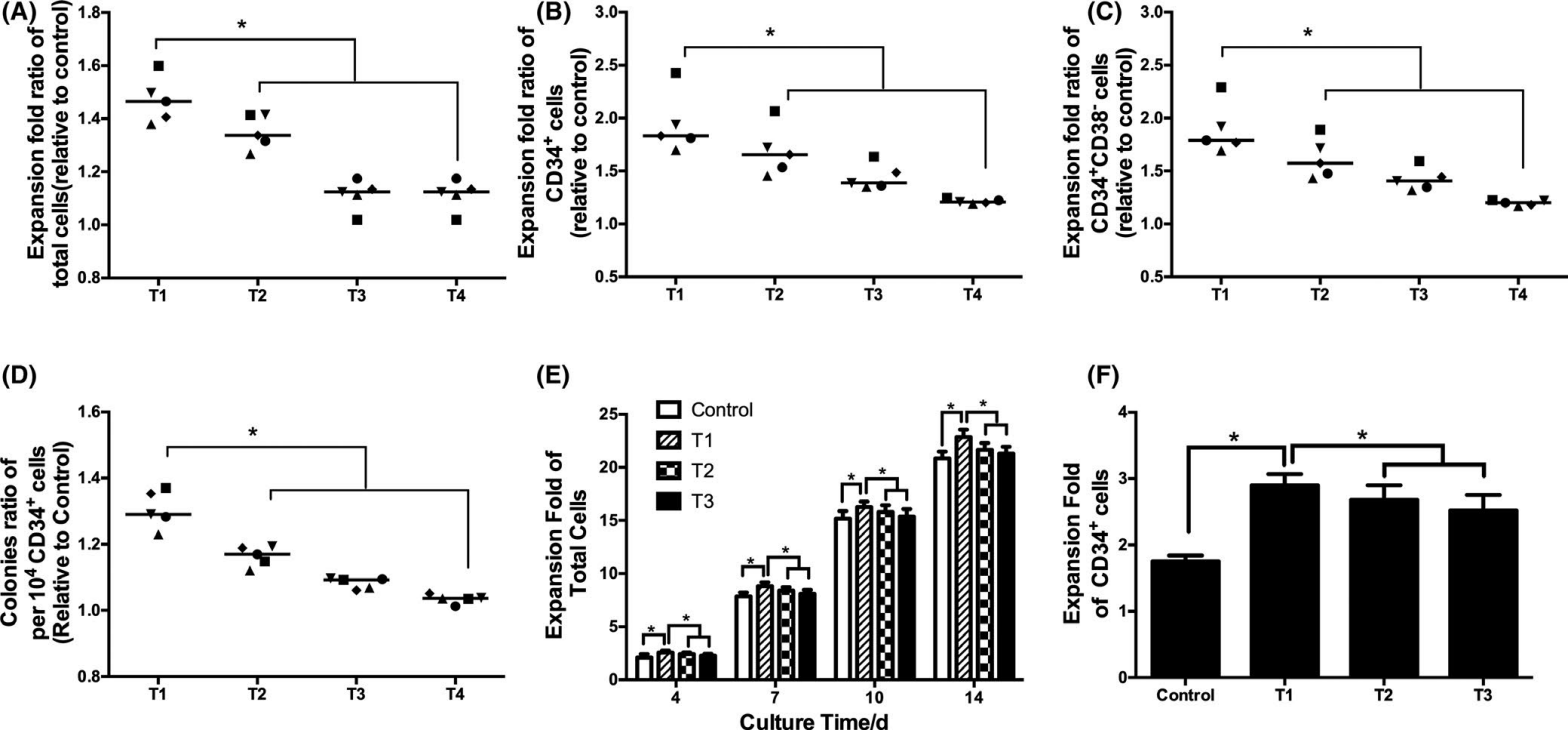
The effect of the SR1 addition strategy on the expansion of HSCs. A, Expansion fold ratio of total cells on day 7. B, Expansion fold ratio of CD34^+^ cells on day 7. C, Expansion fold ratio of CD34^+^CD38^−^ cells on day 7. D, Colonies per 10^4^ CD34^+^ cells. E, Expansion fold of total cells. F, Expansion fold of CD34^+^ cells on day 14. T1: 12 h^−1^ SR1, T2: 24 h^−1^ SR1, T3: 48 h^−1^ SR1, T4: 96 h^−1^ SR1. n = 5, *P < .05

To identify the optimal SR1 addition strategy, SR1 was added as described in Section 2.2. As a result, the relative expansion ratio of total cells, CD34^+^ cells and CD34^+^CD38^−^ cells on day 7 increased with increasing SR1 addition frequency. The T1 group showed the highest relative expansion ratio of total cells, CD34^+^ cells and CD34^+^CD38^−^ cells (Figure 1A‐C). The biological functions of expanded CD34^+^ cells were investigated by colony formation assays and secondary expansion assays. In the semisolid culture, the colony number ratio per 10^4^ CD34^+^ cells increased with increasing SR1 addition frequency, and T1 group‐expanded CD34^+^ cells exhibited the highest number of colony‐forming units (Figure 1D). In secondary expansion culture, T1 group‐expanded CD34^+^ cells presented the highest total cell expansion folds and CD34^+^ cell expansion folds (Figure 1E,F).

**FIGURE 2:**
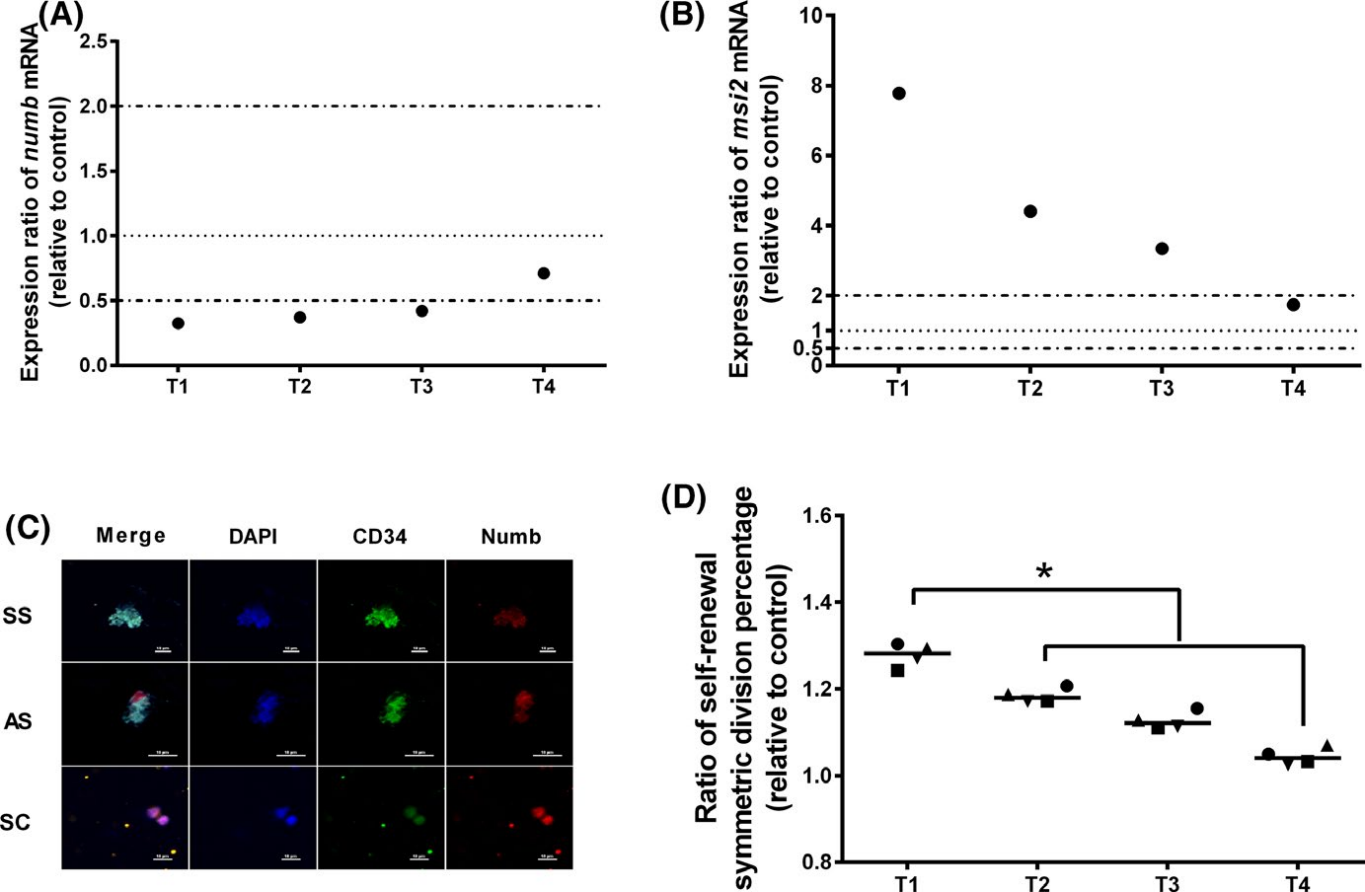
The effect of the SR1 addition strategy on the division mode of CD34^+^ cells. A, mRNA expression of *numb* on day 7. B, mRNA expression of *msi2* on day 7. C, Immunofluorescence staining of HSC division mode. D, ratio of self‐renewal symmetric division percentage on day 7. SS: self‐renewal symmetric division, SD: differentiated symmetric division, AS: asymmetric division. T1: 12 h^−1^ SR1, T2: 24 h^−1^ SR1, T3: 48 h^−1^ SR1, T4: 96 h^−1^ SR1. n = 4, *P < .05

The division mode of CD34^+^ cells was further evaluated. By detection of the key genes *numb* and *msi2* by RT‐PCR, there was a significant downregulation of *numb* and upregulation of *msi2* (Figure 2A,B). By counting cells stained with Numb on day 7, T1 resulted in the highest relative self‐renewal symmetric division percentage ratio of CD34^+^ cells (Figure 2C,D). According to these results, 0.5 μmol/L SR1 addition at a frequency of 12 h^−1^ was the optimal addition strategy.

**FIGURE 3:**
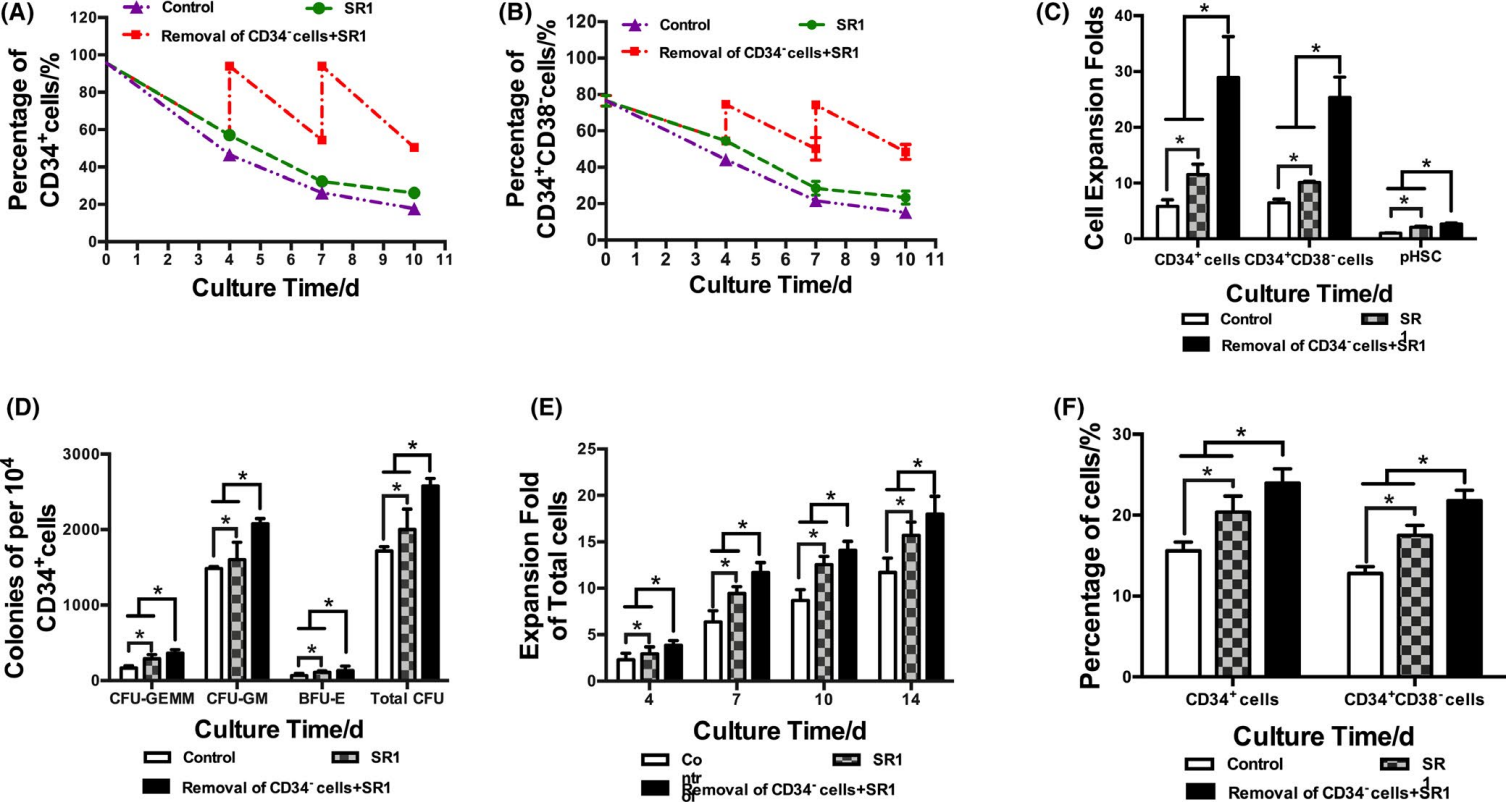
The effect of removal of CD34^−^ cells on the expansion and biological function of CD34^+^ cells. A, Percentage of CD34^+^ cells. B, Percentage of CD34^+^CD38^−^ cells. C, Expansion fold of CD34^+^ cells, CD34^+^CD38^−^ cells and primitive HSCs (pHSCs, CD34^+^CD38^−^CD45RA^−^CD49f^+^CD90^+^ cells). D, Colonies per 10^4^ CD34^+^ cells. E, Expansion fold of total cells. F, Percentage of CD34^+^ cells and CD34^+^CD38^−^ cells. n = 3, *P < .05

To further promote the expansion of HSCs, CD34^−^ cells were periodically removed, and the optimal SR1 addition strategy was applied simultaneously. The expansion fold of CD34^+^ cells and biological function of expanded CD34^+^ cells were explored. The proportion of CD34^+^ cells and CD34^+^CD38^−^ cells was investigated by flow cytometry. The proportion of CD34^+^ cells and CD34^+^CD38^−^ cells decreased continuously in the control group with the extension of culture time. However, the addition of SR1 and removal of CD34^−^ cells with SR1 addition both reduced the decline (Figure 3A,B). The numbers of CD34^+^ cells and CD34^+^CD38^−^ cells were calculated on day 10 (Figure 3C). On day 10, the expansion fold of CD34^+^ cells in the CD34^−^ cell removal plus SR1 group was 28.9 ± 7.3‐fold, significantly higher than the 5.8 ± 1.2‐fold in the control group and 11.5 ± 1.8‐fold in the SR1 group (*P* < .05). The expansion fold of CD34^+^CD38^−^ cells in the CD34^−^ cell removal plus SR1 group was 25.4 ± 3.6‐fold, significantly higher than the 6.5 ± 0.6‐fold in the control group and 10.1 ± 0.2‐fold in the SR1 group (*P* < .05).

It was reported that CD34^+^CD38^−^CD45RA^−^CD49f^+^CD90^+^ cells can be used to characterize human HSPCs.^28^ Thus, the percentages of primitive HSPCs (pHSCs, CD34^+^CD38^−^CD45RA^−^CD49f^+^CD90^+^ cells) were analysed, and the expansion folds of pHSCs were calculated. Higher expansion folds of pHSCs in the CD34^−^ cell removal plus SR1 group were found on day 10 than in the other groups (Figure 3C, *P* < .05).

The biological function of expanded CD34^+^ cells was evaluated by colony‐forming assay and secondary expansion assay (Figure 3D‐F). In the semisolid cultures, the CFU‐GEMM, CFU‐GM, BFU‐E and total CFUs in the CD34^−^ cell removal plus SR1 group were significantly higher than those in the control group and SR1 group (*P* < .05). In secondary expansion cultures, the total cell expansion fold, proportion and expansion fold of CD34^+^ cells and CD34^+^CD38^−^ cells in the CD34^−^ cell removal plus SR1 group were also significantly higher than those in the control group and SR1 group (*P* < .05).

To conclude, removal of CD34^−^ cells with SR1 further promoted the expansion of CD34^+^ cells and maintained the biological function of expanded CD34^+^ cells ex vivo.

**FIGURE 4:**
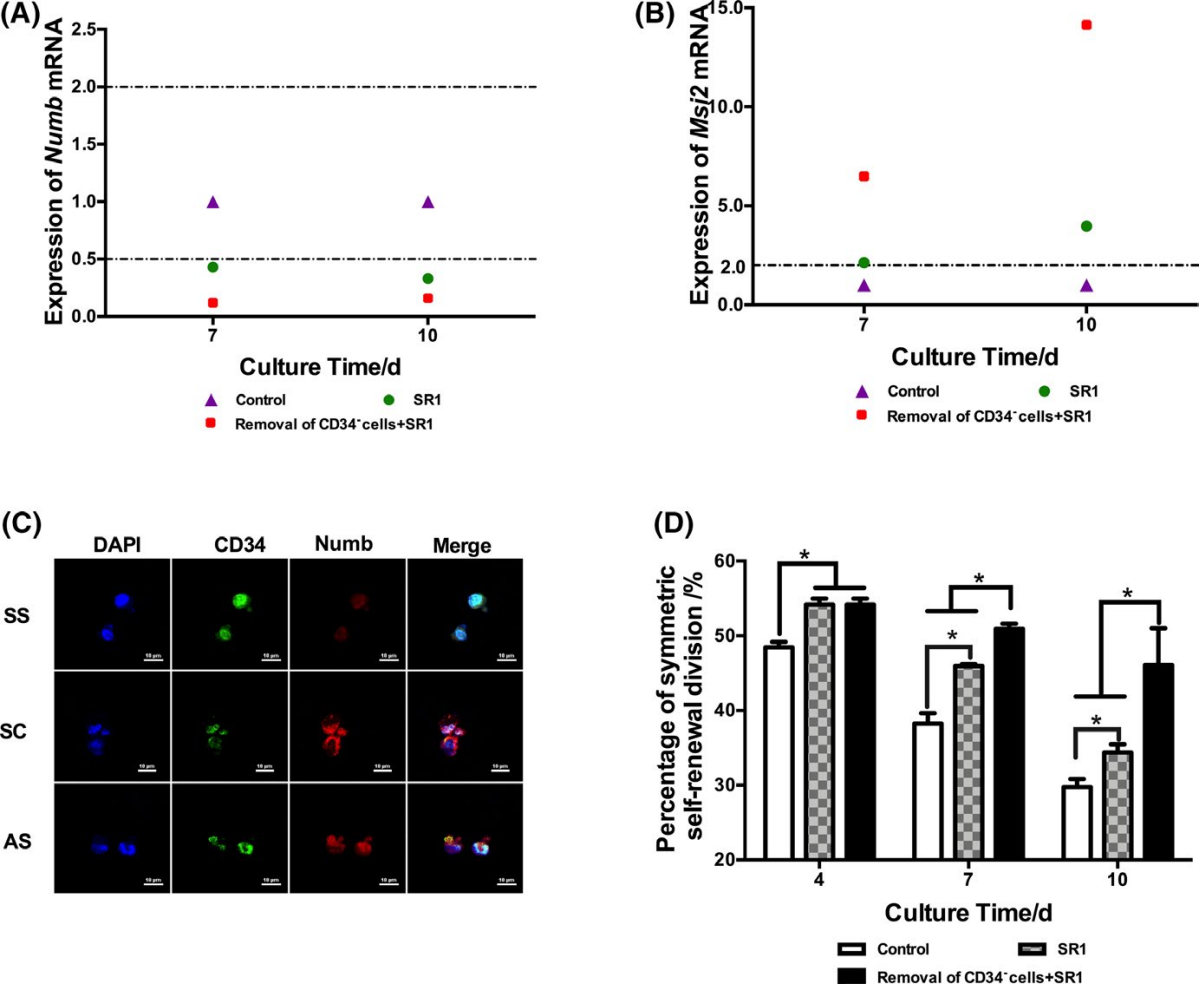
The effect of removal of CD34^−^ cells on the division mode of CD34^+^ cells. A, mRNA expression of *numb*. B, mRNA expression of *msi2*. C, HSC division mode based on immunofluorescence staining. SS: self‐renewal symmetric division, SD: differentiated symmetric division, AS: asymmetric division. D, Percentage of self‐renewal symmetric division of CD34^+^ cells. n = 3, *P < .05

The division mode of CD34^+^ cells was detected (Figure 4). Compared to the control group, the relative mRNA expression of *numb* in the CD34^−^ cell removal plus SR1 group was significantly downregulated relative to that in the control group and SR1 group. The mRNA expression of *msi2* in the CD34^−^ cell removal plus SR1 group was significantly upregulated relative to that of the control group and SR1 group (Figure 4A,B). According to the statistical results of the cell division pattern determined by immunofluorescence staining, the proportions of self‐renewal symmetrical division of CD34^+^ cells in the CD34^−^ cell removal plus SR1 group were significantly higher than those in the control group and SR1 group (Figure 4C,D). These results suggested that with SR1 addition, removal of CD34^−^ cells with SR1 addition increased the proportion of self‐renewal symmetrical division.

### 3.2 Periodic removal of CD34^−^cells reduced the content of TGF‐β1 in supernatant and further downregulated the AhR signalling gene by cooperating with SR1

**FIGURE 5:**
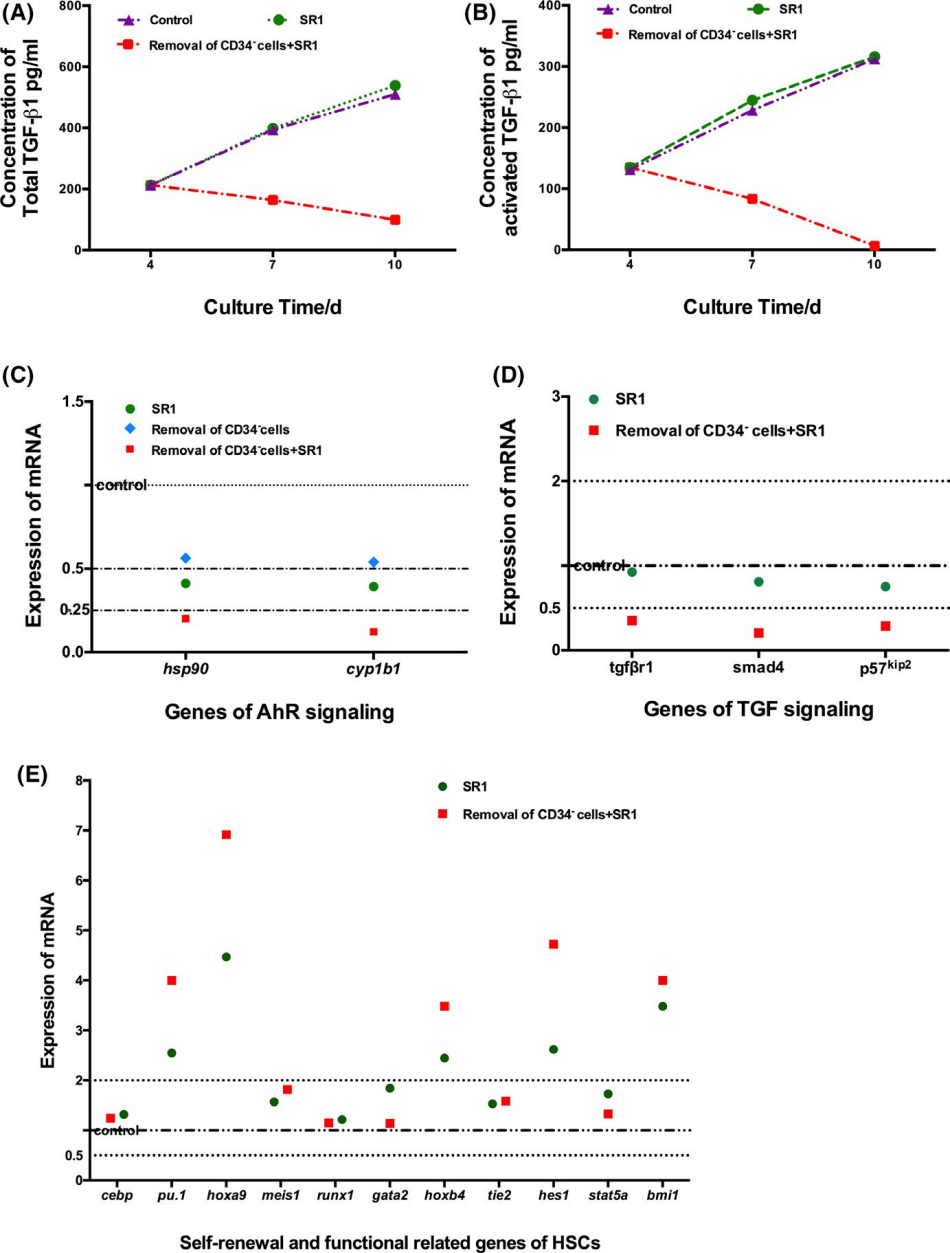
The concentration of TGF‐β1 in the supernatant and the expressions of key genes associated with AhR signalling, TGF‐β signalling, self‐renewal and function of HSCs. A, The concentration of total TGF‐β1. B, The concentration of spontaneously activated TGF‐β1. C, mRNA expression of AhR signalling. D, mRNA expression of TGF‐β signalling. E, mRNA expression of key genes involved in the self‐renewal and function of HSCs. n = 3, *P < .05

By detecting the content of TGF‐β1 in the supernatant (Figure 5A), the results indicated that the content of TGF‐β1 increased with time. In the CD34^−^ cell removal plus SR1 group, the concentration of total TGF‐β1 was significantly reduced to 99.37 ± 31.33 pg/mL on day 10, which was significantly lower than the 510.31 ± 30.55 pg/mL in the control group and 539.14 ± 71.55 pg/mL in the SR1 group (*P* < .05), which showed that periodically removing CD34^−^ cells reduced the TGF‐β1 content. Consistent with total TGF‐β1, the level of spontaneously activated TGF‐β1 in the SR1 group and the CD34^−^ cell removal plus SR1 group were both significantly reduced compared with that of the control group (Figure 5B).

Finally, the expression of the key genes was detected by RT‐PCR (Figure 5C‐E). For the AhR signalling pathway, the expression of *cyp1b1* and *hsp90* was tested. Relative to the control, SR1 addition significantly reduced the expression of *cyp1b1* and *hsp90*. Interestingly, the expression of *cyp1b1* and *hsp90* in the CD34^−^ cell removal plus SR1 group was further decreased, while there was no difference between the control group and the CD34^−^ cell removal group. These results may prompt that removal of CD34^−^ cells that reduced TGF‐β1 cooperation with SR1 further downregulated the expression of the key genes in the AhR signalling pathway (Figure 5C). For TGF signalling, the expression of tgfβr1, smad4 and p57^kip2^ in the CD34^−^ cell removal plus SR1 group was downregulated relative to that of the control (Figure 5D). For self‐renewal and functional genes of HSCs, the expression of *cebp, meis1, runx1, tie2, gata2* and *stat5a* was not significantly different. The expression of *pu.1, hoxa9, hoxb4, hes1* and *bmi1* was upregulated in the SR1 group and the CD34^−^ cell removal plus SR1 group relative to that of the control (Figure 5E).

## 4 DISCUSSION

The clinical application of HSCs has been limited by insufficient quantities of these cells. Many studies have focused on the ex vivo expansion of HSCs to solve the limitation of HSC numbers. It has been reported that inhibition of AhR signalling is an effective way to decrease differentiation. StemRegenin 1 (SR1) is a common inhibitor of the AHR signalling pathway and has been proven to be a positive regulator of the expansion of HSCs.^14^, ^15^, ^16^ Although AHR was initially identified as a receptor for heterogeneous biological compounds, including the environmental toxicant TCDD, numerous studies have identified and identified several endogenous ligands (such as tryptophan derivatives) that may be key components that mediate multiple physiological functions of AhR signalling, most of these endogenous ligands were reported as agonists of AhR signalling.^29^, ^30^, ^31^ Tryptophan and its downstream metabolite indoleacetic acid could be act as direct AhR agonists,^32^ the AhR signalling was activated during ex vivo expansion.^14^ In addition, it was reported that SR1 acted as an antagonist of AhR signalling.^15^ In this work, we also found that SR1 suppressed AhR signalling by downregulating the expression of *cyp1b1* and *hsp90*. HSCs are rich in CD34^+^ cells and inevitably differentiate into CD34^−^ cells during ex vivo expansion. Our previous work found that periodic removal of CD34^−^ cells significantly promoted the expansion of CD34^+^ cells by reducing the TGF‐β1 content. Therefore, to further enhance the expansion of CD34^+^ cells, CD34^−^ cells were periodically removed, and SR1 addition was performed simultaneously. The expansion folds of CD34^+^ cells, CD34^+^CD38^−^ cells and primitive HSCs with periodic removal of CD34^−^ cells and simultaneous SR1 addition were significantly increased. Moreover, the biological function of expanded CD34^+^ cells was better maintained by evaluating their colony‐forming and secondary expansion capabilities. Accurately, the standard evaluation of the biological function of expanded HSCs ex vivo was based on the engraftment ability in NOD/SCID mice. However, the secondary expansion assay and colony‐forming assay have been widely used to evaluate the biological function of expanded HSCs.^33^, ^34^, ^35^ Therefore, the better secondary expansion and colony‐forming abilities of expanded CD34^+^ cells in the CD34^−^ cell removal plus SR1 group maintained better biological function than cells in other groups.

The percentage of self‐renewal symmetric division was also detected, and CD34^+^ cells in the CD34^−^ cell removal plus SR1 group exhibited a significant increase. The division mode of HSCs has been studied for many years.^4^, ^5^, ^7^, ^36^, ^37^, ^38^ Initially, the division mode of HSCs was distinguished by the function of the daughter cells after division.^36^, ^37^ Later, as studies progressed, the division mode of HSCs was detected by the distribution of intracellular proteins in the daughter cells after division.^4^, ^5^, ^7^ To date, the pattern of haematopoietic stem cell division has been based on intracellular proteins, especially changes in the cell division mode when genes encoding these proteins are knocked out or overexpressed.^6^, ^7^, ^38^, ^39^ However, few studies have examined division mode changes of HSCs during ex vivo expansion. It was undoubtedly difficult to detect the division mode of HSCs during ex vivo expansion. However, in our work, the self‐renewal symmetric division percentages of HSCs were not exactly the same. However, these percentages were measured by the distribution of Numb and CD34 under a unified standard. Therefore, to some extent, these factors could be characterized by the self‐renewal symmetric division percentages of HSCs during ex vivo expansion.

TGF‐β1 was identified as an inhibitor of the expansion of HSCs. In our work, the contents of total TGF‐β1 and activated TGF‐β1 were both reduced by removing CD34^−^ cells with SR1 addition. Consistently, the expression of tgfβr1, smad4 and p57^kip2^ in the CD34^−^ cell removal plus SR1 group was also downregulated. TGF‐β1 inhibited haematopoietic function, and its effect depended on its concentration.^19^ TGF‐β1 was found to strongly inhibit the proliferation and differentiation of primitive cells, including CD34^+^CD38^−^ cells and CD34^+^CD38^+^ cells.^22^ SCF‐mediated terminal erythroid differentiation was reduced by neutralization of TGF‐β1 in human cord blood CD34^+^CD38^−^Lin^−^ cells.^40^ Hexachlorobenzene reduced AhR mRNA expression by enhancing TGF‐β1 mRNA levels in human breast cancer cells,^23^ and TGF‐β1 reduced TCDD‐induced AhR gene expression in nontumorigenic prostate epithelial cells.^25^ However, some studies showed that TGF‐β1 stimulated the synthesis of AhR mRNA in human HepG2 cells^27^ and that TGF‐β1 was proven to be an endogenous activator of AHR in a dose‐responsive manner in H1 L7.5c3 cells.^24^ In our work, the expression of the key genes *hsp90* and *cyp1b1* indicated that removing CD34^−^ cells and adding SR1 simultaneously further inhibited the AhR signalling pathway. This demonstrated that TGF‐β1 may neutralize the inhibition of AhR signalling by SR1. However, a verification test with the supplementation of TGF‐β1 and/or neutralizing antibody of TGF‐β1 with SR1 may have been more helpful.

In conclusion, removing CD34^−^ cells and adding SR1 simultaneously reduced the TGF‐β1 content, promoted the expansion of CD34^+^ cells, increased self‐renewal symmetric division and maintained better biological function of expanded CD34^+^ cells by downregulating AhR signalling. These results provide technical support for the optimization of the haematopoietic stem cell culture process ex vivo.

## CONFLICT OF INTEREST

The authors declare no competing financial interests.

## AUTHOR CONTRIBUTIONS

Haibo Cai and Xuejun Zhu conceived the study and designed the experiments. Xuejun Zhu performed the experiment, finished original draft and was responsible for review and editing of manuscript. Qihao Sun contributed to the review and editing of manuscript. Haibo Cai was responsible for supervision, review and editing of manuscript and provided funding for the work. Wen‐Song Tan was responsible for providing funding.

## Data Availability

The data used to support the findings of this study are available from the corresponding author upon request.
